# Biological evaluation of hydroxynaphthoquinones as anti-malarials

**DOI:** 10.1186/1475-2875-12-234

**Published:** 2013-07-10

**Authors:** Desiree C Schuck, Sabrina B Ferreira, Laura N Cruz, David R da Rocha, Miriam S Moraes, Myna Nakabashi, Philip J Rosenthal, Vitor F Ferreira, Celia RS Garcia

**Affiliations:** 1 Departamento de Fisiologia, Universidade de São Paulo, São Paulo 05508-900, Brazil; 2 Departamento de Química Orgânica, Universidade Federal Fluminense, Niterói 24020-141, Brazil; 3 Departamento de Química Orgânica, Universidade Federal do Rio de Janeiro, Macaé 27930-560, Brazil; 4 Department of Medicine, University of California, San Francisco, CA 94143, USA; 5 Universidade de São Paulo, Instituto de Biociências, Rua do Matão, travessa 14, n.321 Cidade Universitária, CEP 05508-900 São Paulo, SP, Brazil

**Keywords:** *Plasmodium falciparum*, Hydroxynaphthoquinone, 2-hydroxy-1,4-naphthoquinone, Mitochondria, Malaria, *Plasmodium berghei*

## Abstract

**Background:**

The hydroxynaphthoquinones have been extensively investigated over the past 50 years for their anti-malarial activity. One member of this class, atovaquone, is combined with proguanil in Malarone®, an important drug for the treatment and prevention of malaria.

**Methods:**

Anti-malarial activity was assessed *in vitro* for a series of 3-alkyl-2-hydroxy-1,4-naphthoquinones (N1-N5) evaluating the parasitaemia after 48 hours of incubation. Potential cytotoxicity in HEK293T cells was assessed using the MTT assay. Changes in mitochondrial membrane potential of *Plasmodium* were measured using the fluorescent dye Mitrotracker Red CMXROS.

**Results:**

Four compounds demonstrated IC_50_s in the mid-micromolar range, and the most active compound, N3, had an IC_50_ of 443 nM. N3 disrupted mitochondrial membrane potential, and after 1 hour presented an IC_50ΔΨmit_ of 16 μM. In an *in vitro* cytotoxicity assay using HEK 293T cells N3 demonstrated no cytotoxicity at concentrations up to 16 μM.

**Conclusions:**

N3 was a potent inhibitor of mitochondrial electron transport, had nanomolar activity against cultured *Plasmodium falciparum* and showed minimal cytotoxicity. N3 may serve as a starting point for the design of new hydroxynaphthoquinone anti-malarials.

## Background

Despite the worldwide effort to understand molecular and cellular features of *Plasmodium falciparum*, the main aetiological agent of human malaria, the disease is still devastating. Parasite resistance to older anti-malarials raises the need for the development of new drugs [1,2]. The anti-malarials currently used stem from six drug classes: aminoquinolines, arylaminoalcohols, artemisinins, antifolates, antibiotics and inhibitors of the respiratory chain [3,4]. The last class is the subject of this report.

The hydroxynaphthoquinones have been extensively investigated over the past 50 years for their anti-malarial activity [5]. Hydrolapachol was the first hydroxynaphthoquinone discovered that possessed anti-malarial activity [6]. This discovery, which emerged at a time of great interest in the study of hydroxynaphthoquinone derivatives as potential new anti-malarials, resulted in the synthesis of a large family of different hydrolapachol analogs [7]. Work on the anti-malarial properties of hydroxynaphthoquinones was revived when chloroquine resistance emerged, and it was discovered that atovaquone effectively inhibits plasmodial electron transport at the ubiquinone (coenzyme Q, 2) site [8].

Atovaquone is a hydroxynaphthoquinone that is used in combination with proguanil (Malarone®) for prophylaxis and therapy of uncomplicated malaria [9]. Atovaquone has excellent anti-malarial activity but exhibits poor pharmaceutical properties, such as low bioavailability and high plasma protein binding [10]. To improve drug bioavailability, several atovaquone analogs were prepared and changes were made to the naphthoquinone moiety, especially the alkyl side chain, because it is known that modifying this chain can alter drug activity [7] and counteract drug resistance [11-13]. Recently, it was demonstrated that 2-methyl-heptyl or 2-methyl-heptyl-trifluoromethyl 2-hydroxy-1,4-naphthoquinones were highly effective against atova-quone-resistant *P. falciparum*[14].

The aim of this work was to test the activity of a new series of hydroxynaphthoquinones [15] against *P. falciparum*. One of these compounds, N3, had an IC_50_ against cultured *P. falciparum* in the nM range, disrupted mitochondrial membrane potential, and had low toxicity against human cells suggesting potential as a lead compound for the development of new anti-malarial agents.

## Methods

### Chemistry

The hydroxynaphthoquinones N1-5 were recently synthesized using a new methodology [15].

### *In vitro* culture of *P. falciparum*

3D7 strain parasites were cultured and synchronized as described previously [16,17]. Briefly, parasites were routinely maintained in A^+^ human erythrocytes (1-3% parasitaemia and 2% haematocrit) in RPMI-1640 media supplemented with 0.2% sodium bicarbonate, 50 mg/L hypoxanthine and 10% type A+ human serum in 92% N_2_, 5% CO_2_ and 3% O_2_.

### Cell culture of HEK293T

HEK293T (human embryonic kidney) cells were cultured in 75 cm^2^ vented tissue culture flasks at 37°C in a humidified atmosphere containing 5% CO_2_ in Dulbecco’s modified essential medium (Gibco BRL) supplemented with 10% (v/v) foetal bovine serum, 100 U/ml penicillin/ and 100 μg/ml streptomycin.

### Flow cytometry analysis

Infected erythrocytes at the ring stage were incubated with different concentrations of the test compounds (N1-N5 0.005, 0.015, 0.045, 0.137, 0.411, 1.23, 3.70, 11.11, 33.33 and 100 μM and atovaquone 0.005, 0.015, 0.045, 0.137, 0.411, 1.23, 3.70, 11.11, 33.33 and 100 nM) for 48 hours; fixed in 2% paraformaldehyde in phosphate-buffered saline (PBS) for 24 hours; permeabilized with 0.1% Triton X-100 and 20 μg/ml RNase; incubated for 30 minutes at 37°C; and stained with 1 nM Yoyo-1 (Molecular Probes). Parasitaemia was determined from dot plots (side scatter versus fluorescence) of 5x10^4^ cells acquired on a FACSCalibur flow cytometer using CELLQUEST software (Becton Dickinson). Initial gating was carried out with unstained, uninfected erythrocytes to account for erythrocyte autofluorescence and analysis performed using Flow Jo 7.6.5 (TreeStar Inc).

### Changes in mitochondrial membrane potential (ΨΔmit)

Loss of parasite mitochondrial membrane potential (ΨΔmit) was determined using 5 μM Mitrotracker Red CMXROS as described previously [18]. Cultures were incubated for 30 min at 37°C with the dye and then for 1 h with 10-fold serial dilutions (0.001-100 μM) of N3 and atovaquone. As a control, 5 μM cyanide *m*-chlorophenylhydrazone (CCCP), a protonophore that dissipate the membrane potential, was used. Results were analysed by flow cytometry as described above.

### Cytotoxicity assays

The toxicity of hydroxynaphthoquinone derivatives toward HEK293T cells was evaluated with the 4,5-dimethylthiazol- 2-yl-2,5- diphenyltetrazolium bromide (MTT) cell proliferation assay [19]. Cells (1.0 × 10^5^/well) were seeded into 48 well plates and incubated in complete medium (Dulbecco’s Modified Eagle Media, GIBCO, Life Technologies; supplemented with 10% foetal bovine serum, 100 μg/ml streptomycin and 100 U/ml penicillin) for 24 h. Thereafter, medium was removed and replaced with complete medium (450 μl/well); N3, atovaquone, and solvent (for controls) were added (0.128, 0.64, 3.2, 16, 80 and 400 μM) and cultures were incubated for 48 hours. Cells were then incubated with the MTT reagent for 3 hours, and absorbance was evaluated.

### Statistical analyses

Analyses of parasitaemia were performed by a one-way analysis of variance test followed by post hoc analysis by the Dunnett’s Multiple Comparison Test using GraphPad Prism software. IC_50_ values were produced using sigmoid dose-response curves on GraphPad software. At least three independent experiments were performed for each assay.

## Results

### *In vitro* activity of new hydroxynaphthoquinones

**Figure 1:**
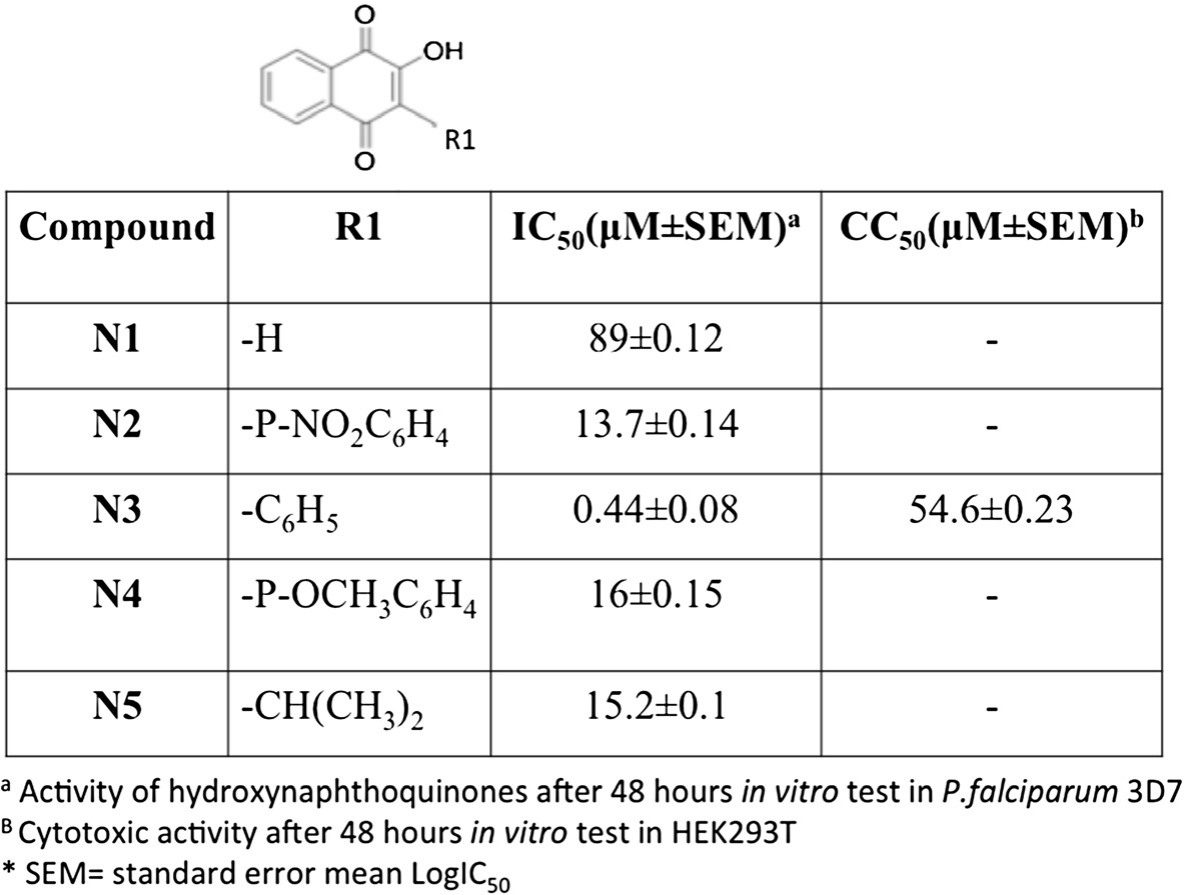
**Biological activity of hydroxynaphtoquinones.** Structure and biological activity of compounds N1-N5.

**Figure 2:**
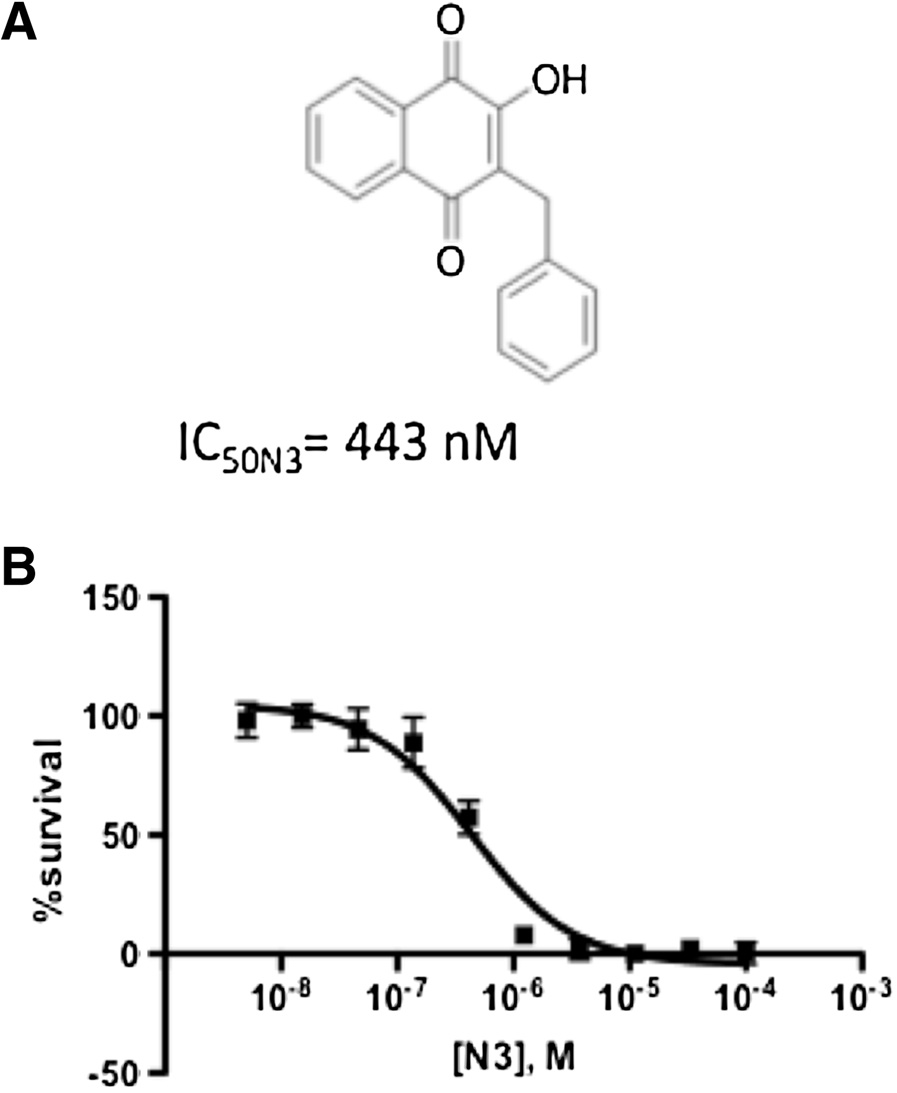
**Effect of new hydroxynaphthoquinones on *P. falciparum *growth. a)** Structure of N3. **b)** Dose response curve for compound N3 incubated for 48 h. Error bars represent standard error of the mean.

The ability of compounds N1-N5 to disrupt the *in vitro* growth of *P. falciparum* was tested. The naphthoquinones showed activity against *P. falciparum*, with IC_50_s of 0.4-89 μM (Figure 1 and Additional file 1: Figure S1). Only N3 had an IC_50_ in the nM range (443 nM; Figure 2).

### Cytotoxicity effects on cells HEK293T

Cytotoxic activity against HEK293T cells was assessed with a tetrazolium-based colorimetric assay. No significant cytotoxicity was observed at concentrations below 16 μM. For N3, the concentration leading to 50% cell death (CC_50_) was 54.6 ± 0.23 μM (Figure 1). For atovaquone the CC_50_ was 49 ± 0.45 μM.

### Effect of N3 on ΔΨ_mit_

**Figure 3:**
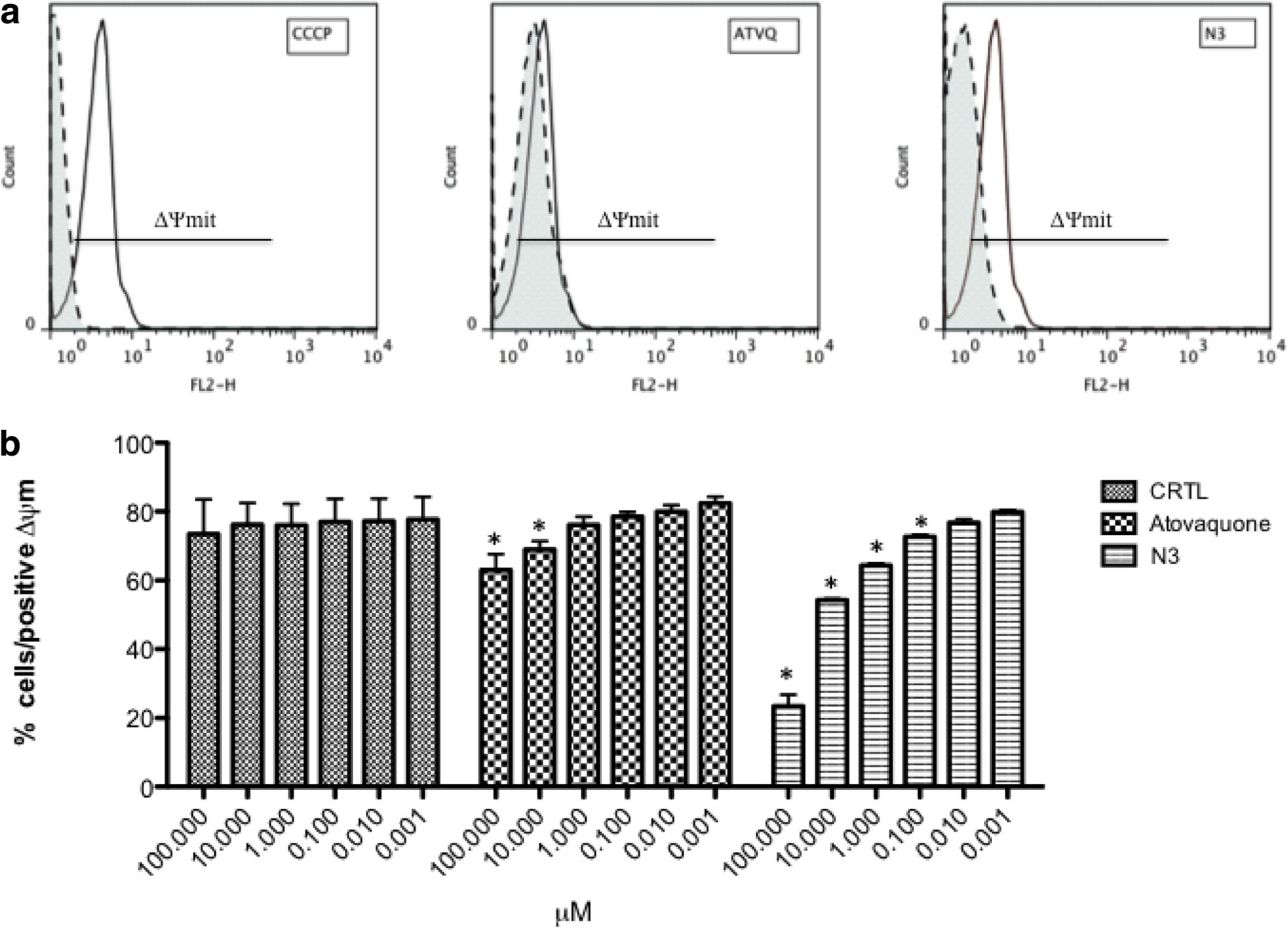
**Proposed mechanism of action of N3. ***P. falciparum* 3D7 was loaded with Mitotracker Red CMXROS and ∆Ψ_Mit_ measured after 1-hour incubations at the indicated concentrations using flow cytometry. **a)** Effects of compounds (dashed lines) compared to untreated controls (solid lines): CCCP 5 μM; N3 100 μM and Atovaquone 100 μM. **b)** Dose responses of different concentrations of solvent control, atovaquone and N3 in the inhibition of ∆Ψ_Mit_. (*) indicates a statistically significant difference from control values (P < 0.05), data were compared using one-way ANOVA and Dunnett’s post test.

It was also verified the effects of compound N3 on *P. falciparum* mitochondrial membrane potential (ΔΨmit). Compound N3 showed an IC_50ΔΨmit_ = 16 μM and atovaquone an IC_50ΔΨmit_ = 4.4 μM (Figure 3).

## Discussion

In an attempt to identify improved anti-malarials, the anti-parasitic activities of synthetic hydroxynaphthoquinones using *in vitro* assays was evaluated. It was identified one compound, N3, with nanomolar activity against *P. falciparum*, confirmed activity against mitochondrial electron transport, and showed limited cytotoxicity against human cells.

The cytochrome bc1 complex catalyses transfer of electrons to maintain the membrane potential of mitochondria, and it is a validated target for anti-malarial drugs. Atovaquone is the only hydroxynaphthoquinone and inhibitor of the bc1 complex currently used to treat malaria. It is generally efficacious, but suffers from irregular absorption (improved with fatty food), limited drug resistance, and high cost of production [8,9,20]. Work to counteract atovaquone limitations has identified other hydroxynaphthoquinones with anti-malarial activity [8]. One series contained an ester at the 3-hydroxy group of atovaquone, with nanomolar anti-malarial activity; addition of long side chains decreased activity [11]. A series of 26 compounds based on the structure of rhinacanthin, a naphthoquinone with anticancer properties, was synthesized [21]; two of these had nanomolar activity and inhibited the cytochrome bc1 complex of *P. falciparum*. Another four hydroxynaphthoquinones were synthesized in an attempt to circumvent resistance to atovaquone, which is mediated by mutations in the mitochondrial cytochrome b gene [13]. The addition of a methyl radical on the naphthoquinone ring provided excellent activity against atovaquone-resistant strains of *P. falciparum*, with documentation of inhibition of the cytochrome bc1 complex [13]. It was recently screened 36 new anti-malarial phenylsulfanylmethyl naphthoquinones structurally related to lapachol [22]. The compounds had moderate *in vitro* activity against *P. falciparum*.

**Figure 4:**
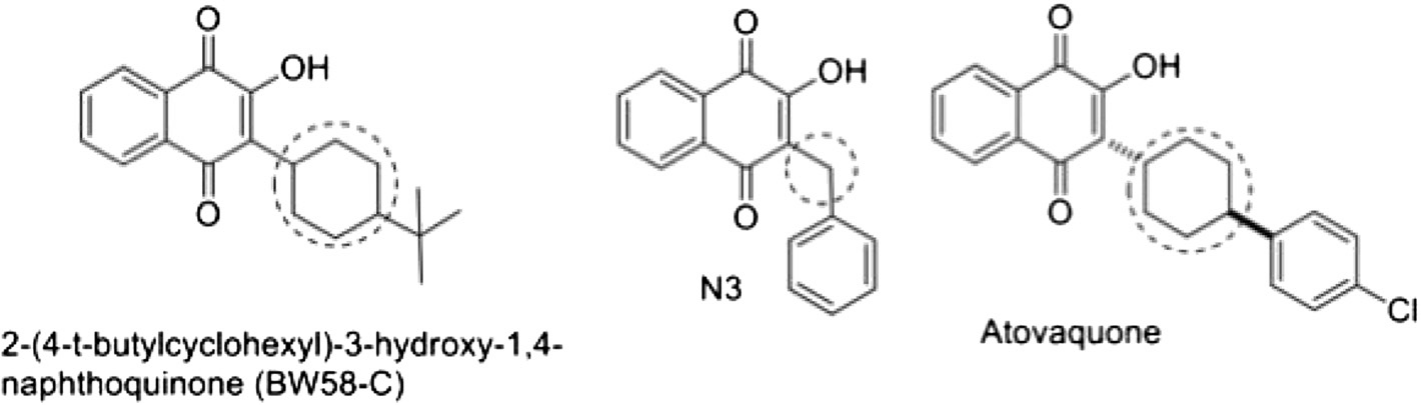
**Structure of BW58-C, N3 and atovaquone pointing the main chemistry differences of compounds.** Circles in the figure represent chiral centers.

Comparing the structures of atovaquone, N3 and BW58-C (an atovaquone precursor), these three structures are very similar in molecular volume, though N3 is much simpler to prepare and has no chiral centers (Figure 4) and, therefore, it can serve as a starting point for a new series of hydroxynaphthoquinone anti-malarials. The results indicate that the cyclohexane ring of atovaquone is not essential for antimalarial activity, since its replacement by a CH2 group in N3 only slightly decreased activity, and N3 was capable of inhibiting mitochondrial activity efficiently (Figures 2 and 3). Considering BW58-C, this molecule showed excellent results against murine malaria [23] and good activity against respiration of mitochondria [24], but it was rapidly metabolized and eliminated in humans [25]. Interaction with cytochrome P450 enzymes and other aspects of metabolism are important components of drug design, and evaluation of the metabolism of N3 is needed.

Screening of a library of 2-hydroxy-naphthoquinones found compounds with alkyl side-chains that effectively inhibited the yeast *b*c1 complex [26]. In the present study, was evaluated 5 additional hydroxynaphthoquinones, and demonstrated that one of these, N3, was a potent inhibitor of mitochondrial electron transport, had nanomolar activity against cultured *P. falciparum*, and showed minimal cytotoxicity. Optimization of N3 thus offers potential for new candidate compounds to treat and prevent malaria.

## Competing interests

The authors declare that they have no competing interests.

## Authors’ contributions

DCS carried out the *in vitro* assays and drafted the manuscript. SBF and DRR carried out the chemical synthesis and collaborated in the elaboration of the manuscript. LNC and MN carried out the cytotoxicity test and collaborated in the elaboration of the manuscript. MN carried out the *Plasmodium* culture and collaborated in the elaboration of the manuscript. CRSG and VFF conceived of the study, and participated in its design and coordination and collaborated in the elaboration of the manuscript. All authors read and approved the final manuscript.

## Supplementary Material

Additional file 1: Figure S1 Structures of other hydroxynaphthoquinones and effects on *P. falciparum* growth. Different concentrations of compounds were incubated for 48 h with *P. falciparum*. Results are shown as a dose response curve for compound N1, N2, N4 and N5 incubated for 48 h. Error bars represent standard error of the mean. Click here for file

## References

[B1] Gazarini ML Sigolo CA Markus RP Thomas AP Garcia CR Antimalarial drugs disrupt ion homeostasis in malarial parasites Mem Inst Oswaldo Cruz 2007 102 329 334 10.1590/S0074-02762007000300012 17568938

[B2] Garcia CR de Azevedo MF Wunderlich G Budu A Young JA Bannister L *Plasmodium* in the postgenomic era: new insights into the molecular cell biology of malaria parasites Int Rev Cell Mol Biol 2008 266 85 156 18544493 10.1016/S1937-6448(07)66003-1

[B3] Schlitzer M Malaria chemotherapeutics part I: History of antimalarial drug development, currently used therapeutics, and drugs in clinical development Chem Med Chem 2007 2 944 986 17530725 10.1002/cmdc.200600240

[B4] WHO World Malaria Report 2010 Geneva: World Health Organization

[B5] Srivastava IK Rottenberg H Vaidya AB Atovaquone, a broad spectrum antiparasitic drug, collapses mitochondrial membrane potential in a malarial parasite J Biol Chem 1997 272 3961 3966 10.1074/jbc.272.7.3961 9020100

[B6] Hooker SC Lomatiol. II. Its occurrence, constitution, relation to and conversion into lapachol. Also a synthesis of lapachol J Am Chem Soc 1936 58 1181 1190 10.1021/ja01298a032

[B7] Fieser LF Berliner E Bondhus FJ Chang FC Dauben WG Ettlinger MG Fields GFM Fieser M Heidelberger C Heymann H Seligman AM Vaughan WR Wilson AG Wilson E Wu M Leffler MT Hamlin KE Hathaway RJ Matson EJ Moore EE Moore MB Rapala RT Zaugg HE Naphthoquinone antimalarials.4.5.6.7.8.9.10.11. synthesis J Am Chem Soc 1948 70 3174 3215 10.1021/ja01190a004 18891815

[B8] Barton V Fisher N Biagini GA Ward SA O’Neill PM Inhibiting *Plasmodium* cytochrome bc1: a complex issue Curr Opin Chem Biol 2010 14 440 446 10.1016/j.cbpa.2010.05.005 20570550

[B9] Baggish AL Hill DR Antiparasitic agent atovaquone Antimicrob Agents Chemother 2002 46 1163 1173 10.1128/AAC.46.5.1163-1173.2002 11959541 PMC127192

[B10] Dressman JB Reppas C *In vitro-in vivo* correlations for lipophilic, poorly water-soluble drugs Eur J Pharm Sci 2000 11 Suppl 2 S73 S80 11033429 10.1016/s0928-0987(00)00181-0

[B11] Danoun S Baziard-Mouysset G Stigliani J Ane-Margail M Payard M Leger J Canron X Vial H Loiseau P Bories C Recoche C Synthesis and protozoocidal activity of new 1,4-naphthoquinones Heterocycl Comm 1999 5 343 348

[B12] El Hage S Ane M Stigliani J Marjorie M Vial H Baziard-Mouysset G Payard M Synthesis and antimalarial activity of new atovaquone derivatives Eur J Med Chem 2009 44 4778 4782 10.1016/j.ejmech.2009.07.021 19747753

[B13] Hughes L Lanteri C O’Neil M Johnson J Gribble G Trumpower B Design of anti-parasitic and anti-fungal hydroxy-naphthoquinones that are less susceptible to drug resistance Mol Biochem Parasitol 2011 177 12 19 10.1016/j.molbiopara.2011.01.002 21251932 PMC5054302

[B14] Hughes L Covian R Gribble G Trumpower B Probing binding determinants in center P of the cytochrome bc(1) complex using novel hydroxy-naphthoquinones Biochim Biophys Acta 2010 1797 38 43 19660431 10.1016/j.bbabio.2009.07.010 PMC2787711

[B15] Ferreira S da Rocha D Carneiro J Santos W Ferreira V A new method to prepare 3-Alkyl-2-hydroxy-1,4-naphthoquinones: synthesis of lapachol and phthiocol Synlett 2011 2011 1551 1554 10.1055/s-0030-1260771

[B16] Trager W Jensen J Human malaria parasites in continuous culture Science 1976 193 673 675 10.1126/science.781840 781840

[B17] Lambros C Vanderberg J Synchronization of *Plasmodium falciparum* erythrocytic stages in culture J Parasitol 1979 65 418 420 10.2307/3280287 383936

[B18] Jogdand PS Singh SK Christiansen M Dziegiel MH Singh S Theisen M Flow cytometric readout based on Mitotracker Red CMXRos staining of live asexual blood stage malarial parasites reliably assesses antibody dependent cellular inhibition Malar J 2012 11 235 10.1186/1475-2875-11-235 22818754 PMC3418546

[B19] Carmichael J DeGraff WG Gazdar AF Minna JD Mitchell JB Evaluation of a tetrazolium-based semiautomated colorimetric assay: assessment of chemosensitivity testing Cancer Res 1987 47 936 942 3802100

[B20] Kessl JJ Meshnick SR Trumpower BL Modeling the molecular basis of atovaquone resistance in parasites and pathogenic fungi Trends Parasitol 2007 23 494 501 10.1016/j.pt.2007.08.004 17826334

[B21] Kongkathip N Pradidphol N Hasitapan K Grigg R Kao WC Hunte C Fisher N Warman AJ Biagini GA Kongsaeree P Chuawong P Kongkathip B Transforming rhinacanthin analogues from potent anticancer agents into potent antimalarial agents J Med Chem 2010 53 1211 1221 10.1021/jm901545z 20067272

[B22] Sharma A Santos I Gaur P Ferreira V Garcia C Rochab D Addition of thiols to o-quinone methide: New 2-hydroxy-3-phenylsulfanylmethyl [1,4]naphthoquinones and their activity against the human malaria parasite *Plasmodium falciparum* (3D7) Eur J Med Chem 2013 59 48 53 23202850 10.1016/j.ejmech.2012.10.052

[B23] Hudson A Randall A Fry M Ginger C Hill B Latter V McHardy N Williams R Novel anti-malarial hydroxynaphthoquinones with potent broad spectrum anti-protozoal activity Parasitology 1985 90 45 55 10.1017/S0031182000049003 3920634

[B24] Hammond D Burchell J Pudney M Inhibition of pyrimidine biosynthesis de novo in *Plasmodium falciparum* by 2-(4-t-butylcyclohexyl)-3-hydroxy-1,4-naphthoquinone in vitro Mol Biochem Parasitol 1985 14 97 109 10.1016/0166-6851(85)90109-4 3885032

[B25] Weaver R Dickins M Burke M Cytochrome P450 2C9 is responsible for hydroxylation of the naphthoquinone antimalarial drug 58C80 in human liver Biochem Pharmacol 1993 46 1183 1197 10.1016/0006-2952(93)90467-B 8216369

[B26] Kessl JJ Moskalev NV Gribble GW Nasr M Meshnick SR Trumpower BL Parameters determining the relative efficacy of hydroxy-naphthoquinone inhibitors of the cytochrome bc1 complex Biochim Biophys Acta 2007 1767 319 326 10.1016/j.bbabio.2007.02.014 17383607 PMC1939967

