# Supplementary material for: Biological evaluation of hydroxynaphthoquinones as anti-malarials
Source: Malar J. 2013 Jul 10;12:234. doi: 10.1186/1475-2875-12-234 (PMC3726445; doi:10.1186/1475-2875-12-234)
Supplement: Additional file 1: Figure S1 — Structures of other hydroxynaphthoquinones and effects on P. falciparum growth. Different concentrations of compounds were incubated for 48 h with P. falciparum. Results are shown as a dose response curve for compound N1, N2, N4 and N5 incubated for 48 h. Error bars represent standard error of the mean. [file 1475-2875-12-234-S1.docx]

**Figure S1**


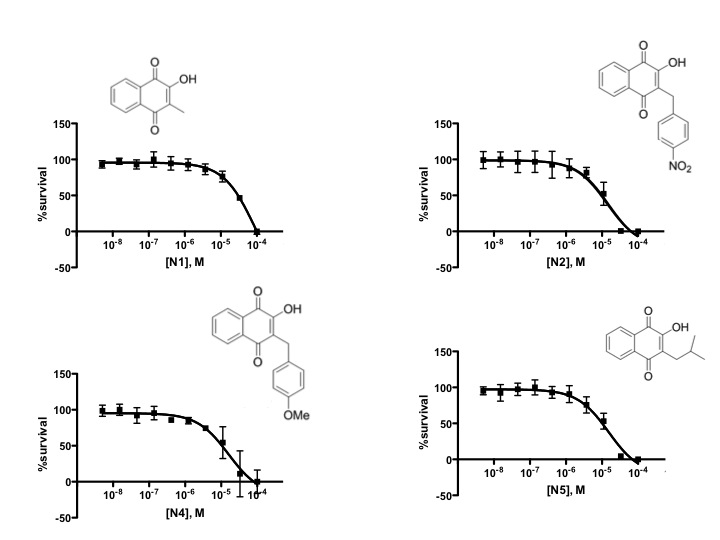


**Figure S1.** Structures of other hydroxynaphthoquinones and effects on on *P. falciparum* growth. Different concentrations of compounds were incubated for 48h with *P. falciparum*. Results are shown as a dose response curve for compound N1, N2, N4 and N5 incubated for 48h. Error bars represent standard error of the mean.
